# C-857-05. Data After Death: Post-mortem Ethics of Burn Patient Records and Images

**DOI:** 10.1093/jbcr/irag033.105

**Published:** 2026-04-06

**Authors:** Joshua Khorsandi, Liahm Blank, Samir Alkhouri, Abu-Bakr Ahmed, Kathryn Cataldo

**Affiliations:** Kirk Kerkorian School of Medicine at University of Nevada, Las Vegas, NV; Kirk Kerkorian School of Medicine at University of Nevada, Las Vegas, NV; Kirk Kerkorian School of Medicine at University of Nevada, Las Vegas, NV; Kirk Kerkorian School of Medicine at University of Nevada, Las Vegas, NV; Department of Plastic and Reconstructive Surgery, University of Nevada, Las Vegas, NV

## Abstract

**Introduction:**

Burn care frequently relies on extensive documentation, including graphic photographic images and detailed clinical records. While these materials are essential for diagnosis, treatment planning, and research, their use after a patient’s death raises complex ethical questions. The emergence of new technologies such as artificial intelligence training, alongside the increased visibility of burn images in education and public health campaigns, challenges traditional notions of confidentiality and consent. This narrative review examines the ethical boundaries of using burn patient records and images post-mortem, with a focus on emerging concerns around digital remains and posthumous consent.

**Methods:**

A narrative review of peer-reviewed literature, professional guidelines, and position statements published between 2000 and 2025 was conducted. Sources included PubMed, Scopus, and Google Scholar, with search terms such as “burn injuries,” “medical photography,” “post-mortem consent,” “digital remains,” and “medical ethics.” Relevant publications addressing clinical practice, teaching, research, and social media use in burn care were synthesized to identify key ethical themes and gaps.

**Results:**

The literature reveals that while ethical frameworks for consent, privacy, and confidentiality are well established during life, guidance becomes inconsistent once the patient has died. A small but growing body of scholarship highlights posthumous privacy as an emerging domain of bioethics. Across studies, concerns included dignity after death, risks of re-identification on digital platforms, and the absence of explicit patient directives regarding posthumous use of images and data. Current medical guidelines provide minimal direction, leaving significant ambiguity for clinicians and researchers.

**Conclusions:**

The ethical use of burn patient images and records after death is an underexplored area, particularly in the context of AI training datasets and social media awareness campaigns. The absence of consensus underscores the need for professional societies to establish clearer policies.

**Applicability to Practice:**

By raising awareness of post-mortem digital ethics, this review emphasizes the importance of developing protocols that honor patient dignity beyond life. Establishing standards for posthumous consent will help clinicians, educators, and researchers navigate the evolving landscape of digital medicine responsibly.

